# mTORC1 signalling is not essential for the maintenance of muscle mass and function in adult sedentary mice

**DOI:** 10.1002/jcsm.12505

**Published:** 2019-11-07

**Authors:** Alexander S. Ham, Kathrin Chojnowska, Lionel A. Tintignac, Shuo Lin, Alexander Schmidt, Daniel J. Ham, Michael Sinnreich, Markus A. Rüegg

**Affiliations:** ^1^ Biozentrum University of Basel Basel Switzerland; ^2^ Department of Biomedicine, Pharmazentrum University of Basel Basel Switzerland; ^3^ Proteomics Core Facility, Biozentrum University of Basel Basel Switzerland

**Keywords:** Raptor, Muscle atrophy, Protein translation, Fibre‐type, TOP mRNA

## Abstract

**Background:**

The balance between protein synthesis and degradation (proteostasis) is a determining factor for muscle size and function. Signalling via the mammalian target of rapamycin complex 1 (mTORC1) regulates proteostasis in skeletal muscle by affecting protein synthesis and autophagosomal protein degradation. Indeed, genetic inactivation of mTORC1 in developing and growing muscle causes atrophy resulting in a lethal myopathy. However, systemic dampening of mTORC1 signalling by its allosteric inhibitor rapamycin is beneficial at the organismal level and increases lifespan. Whether the beneficial effect of rapamycin comes at the expense of muscle mass and function is yet to be established.

**Methods:**

We conditionally ablated the gene coding for the mTORC1‐essential component raptor in muscle fibres of adult mice [inducible raptor muscle‐specific knockout (iRAmKO)]. We performed detailed phenotypic and biochemical analyses of iRAmKO mice and compared them with muscle‐specific raptor knockout (RAmKO) mice, which lack raptor in developing muscle fibres. We also used polysome profiling and proteomics to assess protein translation and associated signalling in skeletal muscle of iRAmKO mice.

**Results:**

Analysis at different time points reveal that, as in RAmKO mice, the proportion of oxidative fibres decreases, but slow‐type fibres increase in iRAmKO mice. Nevertheless, no significant decrease in body and muscle mass or muscle fibre area was detected up to 5 months post‐raptor depletion. Similarly, *ex vivo* muscle force was not significantly reduced in iRAmKO mice. Despite stable muscle size and function, inducible raptor depletion significantly reduced the expression of key components of the translation machinery and overall translation rates.

**Conclusions:**

Raptor depletion and hence complete inhibition of mTORC1 signalling in fully grown muscle leads to metabolic and morphological changes without inducing muscle atrophy even after 5 months. Together, our data indicate that maintenance of muscle size does not require mTORC1 signalling, suggesting that rapamycin treatment is unlikely to negatively affect muscle mass and function.

## Introduction

Maintaining skeletal muscle is crucial for preserving the quality of life. Skeletal muscle atrophy, which occurs as a consequence of a range of conditions and diseases, such as immobilization, cancer cachexia, or sarcopenia, the age‐related loss of muscle mass and function, decreases independence and increases the risk of morbidity and mortality.^1^, ^2^ Understanding the underlying mechanisms involved in maintaining skeletal muscle is a pre‐requisite for developing effective therapeutic strategies to prevent muscle atrophy. A protein complex postulated to be a key regulator in maintaining skeletal muscle mass is the mammalian (or mechanistic) target of rapamycin complex 1 (mTORC1).^3^ mTORC1 consists of the serine/threonine kinase mTOR, the regulatory‐associated protein of mTOR (raptor), which is essential for the stability and function of the complex and the mammalian lethal with Sec13 protein 8.^4^ Together, these proteins sense adenosine triphosphate, amino acid, and insulin levels to promote or suppress protein translation.^4^ mTORC1 promotes translation by releasing the eukaryotic translation initiation factor 4E (eIF4E) from its inhibitory binding partner eIF4E‐binding protein 1 (4E‐BP1),^5^ thereby allowing the formation of the eIF4F complex and recruitment of the pre‐initiation complex to the 5′ end of mRNA.^6^ Additionally, mTORC1 phosphorylates and activates ribosomal protein S6 kinase 1 that in turn phosphorylates S6, elongation factor 2, and initiation factor 4B to promote protein synthesis.^6^ Genetic or pharmacological inhibition of mTORC1 strongly decreases mRNA translation.^7^ In skeletal muscle, pharmacological inhibition or genetic ablation of crucial components of mTORC1 prevents overload‐induced hypertrophy in rodents^8^, ^9^, ^10^, and the mTORC1 inhibitor rapamycin blunts the amino acid‐induced increase in protein synthesis in humans.^11^, ^12^ Furthermore, muscle fibre‐specific knockout of raptor^13^ or mTOR^14^ in developing mice reduces body and muscle mass, causes severe myopathic features, and strongly reduces lifespan. Finally, ablation of raptor during myogenesis largely abrogates the formation of skeletal muscle.^15^ These results clearly demonstrate the importance of mTORC1 in skeletal muscle development and growth.

Based on these results, mTORC1 is also assumed to be a key regulator of muscle maintenance.^3^ However, previous genetic studies that abrogated mTORC1 function were conducted during muscle growth^8^, ^9^, ^10^ or used developmental knockout models^13^, ^14^, ^15^ where the phenotype may be the result from impairments during the rapid postnatal growth phase. Interestingly, long‐term rapamycin treatment of adult mice does not appear to negatively affect whole‐body muscle function.^16^, ^17^ The interpretation of these experiments is difficult, however, as long‐term rapamycin treatment also affects mTORC2 function^18^ and because rapamycin only partially blocks mTORC1.^19^ Therefore, the importance of mTORC1 in muscle maintenance is still unanswered. To analyse the role of mTORC1 specifically during adult muscle maintenance, we developed an inducible mouse model using the Tamoxifen (TAM)/Mer‐Cre‐Mer system^20^ where the gene for raptor (*Rptor*) can be ablated specifically in skeletal muscle during adulthood. By thoroughly examining the phenotype of these mice, from 10 days up to 5 months after raptor depletion, we demonstrate that, while these mice phenocopy some aspects of constitutive mTORC1 depletion in skeletal muscle, muscle mass and function are not affected for up to 5 months post‐depletion. These results strongly indicate that muscle maintenance in sedentary mice is largely independent of mTORC1 activity and provide another example of the complex and context‐dependent requirements of mTORC1 function.

## Methods

### Mice

All mice were kept under a 12 h light to dark cycle in the animal facility of the Biozentrum (Basel, Switzerland) and received *ad libitum* access to water and standard laboratory chow. All experiments were approved by the veterinary commission of the Canton Basel‐Stadt (Schlachthofstrasse 55, 4056 Basel, Switzerland).

TAM‐inducible, muscle‐specific raptor knockout mice (iRAmKOs) were generated by crossing three mouse lines. The mouse line with muscle‐specific expression of Cre recombinase fused to two mutated oestrogen receptors human skeletal actin [(*HSA)–MerCreMer*] was generated and provided by Dr K. Esser. 20 The modified *Rosa26* promoter with enhanced green fluorescent protein (EGFP) as a Cre reporter gene (*mR26–pCAG–EGFP* ^*ki/ki*^) was generated and provided by Dr M. Mueller.^21^ Homozygous *Rptor exon 6* floxed mice (*Rptor* ^*fl/fl*^) were developed by our laboratory as previously described.^13^ Experimental iRAmKO mice (*HSA–Mer–Cre–Mer* ^*wt/ki*^; *Rptor* ^*fl/fl*^; and *mR26–pCAG–EGFP* ^*ki/ki*^) and controls were generated by crossing *Rptor* ^*fl/fl*^ and *mR26–pCAG–EGFP* ^*ki/ki*^ with iRAmKO mice. *HSA–Mer–Cre–Mer* ^*wt/ki*^‐positive offspring were used as experimental mice (termed iRAmKOs), while Cre‐negative mice were used as controls. To determine whether the mice were positive or negative for Cre, we genotyped a toe‐snip with forward (TGT GGC TGA TGA TCC GAA TA) and reverse primers (GCT TGC ATG ATC TCC GGT AT). All iRAmKOs and control mice were male.

### Tamoxifen administration

TAM powder (Sigma‐Aldrich, St. Louis, MO, USA) was dissolved in corn oil (Sigma‐Aldrich) at a concentration of 20 mg/mL. TAM was administrated via intraperitoneal injections of 100 μL when the mice were between 12 and 16 weeks old. The first administration regime comprised of five injections on five consecutive days (Supporting Information, *Figure* *S1* *A*). The day after the fifth injection was defined as Day 1. TAM was administered again on Days 3 and 4. For prolonged knockouts, two additional injections were administered on consecutive days every 28 days to prevent potential reintroduction of raptor into fibres by non‐targeted muscle stem cells.

### Sample preparation and western blot analysis

Muscles were powdered on a metal block cooled with liquid nitrogen. The tissue was lyzed in cold radioimmunoprecipitation assay buffer (50 mM Tris‐Base, 150 mM NaCl, 0.5% sodium deoxycholate, 0.1% SDS, and 1% NP‐40, all at pH 8) supplemented with a phosphatase (Roche, Basel, Switzerland) and protease inhibitor cocktail (Roche). The samples were incubated on a rotating wheel for 2 h at 4°C, sonicated twice for 8 s, and centrifuged at 15 700 rcf for 30 min at 4°C. The protein concentration was measured on the cleared lysates with Pierce™ BCA Protein Assay Kit (Thermo Fisher Scientific, Waltham, MA, USA). After diluting the lysates in radioimmunoprecipitation assay and Laemmli buffer, equal protein amounts were loaded onto SDS gels (NuPage 4–12% Bis‐Tris). After electrophoretic separation, proteins were transferred onto a nitrocellulose membrane on ice at 100 V for 1 h and stained with Ponceau to cut at the appropriate heights. The membrane slices were blocked with 4% (w/v) bovine serum albumin in TBS‐T solution (0.137 M NaCl, 2.68 mM KCl, 0.025 M‐Tris‐HCl pH 7.4, 0.1% Tween‐20) for 45 min at room temperature (RT). The primary antibodies were incubated over night at 4°C. After a washing step with TBS‐T, the secondary antibodies were added for 1 h at RT. Western blot detection was done using the KLP LumiGlo Chemiluminescence Substrate Kit (Seracare, Milford, MA, USA) and imaged in a Fusion Fx machine (Vilber, Collégien, France). α‐Actinin was used as a housekeeping gene as well as for normalization.

### Antibodies used in western blot

The following primary antibodies from Cell Signalling Technology (Danvers, MA, USA) were used: 4E‐BP1 (#9452), P‐4E‐BP1^S65^ (#9451), AKT (#9272), P‐AKT^S473^ (#9271), P‐AKT^T308^ (#9275), eIF4E (#2067), mTOR (#2972), P‐mTOR^S2448^ (#2971), raptor (#2280), S6 (#2217), and S6^S240/244^ (#5364). The primary antibody for α‐actinin (#A7732) was purchased from Abcam (Cambridge, England). The primary antibody for EGFP (11814460001) was from Roche Applied Science (Penzberg, Germany). All Cell Signalling Technology antibodies as well as EGFP were diluted 1:1000; α‐actinin was diluted 1:5000. The secondary antibodies goat anti‐mouse (#115‐035‐003) and goat anti‐rabbit (#111‐035‐003) were purchased from Jackson ImmunoResearch Europe (Ely, Cambridgeshire, England) and were diluted 1:10 000. All antibodies were diluted in 4% bovine serum albumin in TBS‐T.

### Immunohistochemistry

The muscles were frozen in 2‐methylbutan directly after removal. All cross‐sections were prepared on a cryostat at a thickness of 10 μm and stored at −20°C. For fibre‐type staining, the sections were first rehydrated with phosphate‐buffered saline (PBS) and then blocked for 30 min with blocking solution [0.4% Triton X‐100, 10% goat serum (Biological industries, Beit Haemek, Israel) in PBS] followed by a washing step with PBS. The primary antibodies against MyHC 2b (1:100; #BF‐F3; DSHB, Iowa, USA), MyHC 2a (1:200; #SC‐71; DSHB), MyHC 1 (1:50; BA‐D5; DSHB), and laminin (1:160; #ab11575; Abcam) were diluted in PBS containing 10% goat serum (Biological industries) and added for 1 h at RT. After a washing step with PBS, the secondary antibodies were added containing AF488 Gt anti‐Ms IgM (1:100; Invitrogen, Carlsbad, CA, USA), AF568 Gt anti‐Ms IgG1 (1:100; Invitrogen), DL405 Gt anti‐Ms IgG2b (1:50; Jackson), and AF647 Donkey anti‐Rabbit IgG (1:200; Jackson) diluted in PBS containing 10% goat serum. The slides were again washed with PBS and then mounted with Vectashield Hardset (H‐1600; Vector Labs, Burlingame, CA, USA). The entire section was imaged on a Zeiss (Oberkochen, Germany) Axio Scan.Z1 Slide Scanner.

### Real‐time quantitative polymerase chain reaction

Total RNA was isolated from the soleus using the Qiagen (Hilden, Germany) RNeasy Mini Kit. 500 ng of RNA was then used to generate first strand complimentary DNA (cDNA) using the iScript cDNA Synthesis Kit (Bio‐Rad, Hercules, CA, USA). The cDNA was diluted 1:10 before usage and amplified on a LightCycler 480 (Roche Diagnostics, Basel, Switzerland) using the Applied Biosystems SYBR Green Master Mix (Roche) on a 384‐well plate. The data was analysed and quantified using the delta delta Ct method 22. β‐Actin was used as a housekeeping gene as well as for normalization. The following primers were used: *Actb* fw: CAG CTT CTT TGC AGC TCC TT and bw: GCA GCG ATA TCG TCA TCC A; *Cox5b* fw: CTT CAG GCA CCA AGG AAG AC and bw: TTC ACA GAT GCA GCC CAC TA; *Cycs* fw: AAA TCT CCA CGG TCT GTT CG and bw: TAT CCT CTC CCC AGG TGA TG; *Fbxo32* fw: CTC TGT ACC ATG CCG TTC CT and bw: GGC TGC TGA ACA GAT TCT CC; *Mb* fw: ATC CAG CCT CTA GCC CAA TC and bw: GAG CAT CTG CTC CAA AGT CC; *Ppargc1α* fw: TGA TGT GAA TGA CTT GGA TAC AGA CA and bw: GCT CAT TGT TGT ACT GGT TGG ATA TG; *Trim63* fw: ACC TGC TGG TGG AAA ACA and bw: AGG AGC AAG TAG GCA CCT CA.

### Muscle force measurements

The *ex vivo* force measurement of the *extensor digitorum longus* (EDL) and the soleus was done as described previously.^23^ Briefly, muscles were carefully excised and mounted on the 1200A Isolated Muscle System (Aurora Scientific, Aurora, ON, Canada) in an organ bath containing 60 mL of Ringer solution (137 mM NaCl, 24 mM NaHCO_3_, 11 mM Glucose, 5 mM KCl, 2 mM CaCl_2_, 1 mM MgSO_4_, and 1 mM NaH_2_PO_4_) that was gassed with 95% O_2_–5% CO_2_ at 30^o^C. Specific forces are derived by normalizing to the cross‐sectional area as described.^24^ The fatigue of EDL and soleus muscles was assessed by 6 min stimulation at 200 Hz and 120 Hz, respectively.

### 7‐Methylguanosine 5′‐triphosphate pulldown

The gastrocnemius was powdered on liquid nitrogen and lysed for 1 h at 4°C in lysis buffer (20 mM Hepes (pH 7.4), 50 mM KCl, 0.2 mM EDTA, 25 mM β‐glycerophosphate, 0.5 mM sodium‐orthovanadate, 1 mM dithiothreitol, 0.5% Triton X‐100, and 50 mM NaF) supplemented with a protease inhibitor cocktail tablet (Roche). After additional cell disruption with a potter homogenizer, the lysate was centrifuged for 10 min at 9800 rcf at 4°C. The protein concentration was measured using the Pierce™ BCA Protein Assay Kit (Thermo Fisher Scientific). A total of 400 μg was used for the pulldown. For pre‐clearance, 15 μL of blank Agarose beads (Jena Bioscience, Thüringen, Germany) were added for 30 min at 4°C. The samples were then centrifuged for 10 min at 9800 rcf at 4°C. Next, 20 μL of a 50% slurry of 7‐methylguanosine 5′‐triphosphate (m7GTP)‐Agarose beads (Jena Bioscience) was added to the supernatant overnight at 4°C. The beads were then washed three times using 200 μL of the lysis buffer for 10 min at 9800 rcf. To finish, the samples were denatured in Laemmli buffer at 95°C before loading them for western blot analysis.

### Fibre area and fibre size distribution

The fibre size distribution and the fibre‐type composition were evaluated using the fibre‐type staining described under Immunohistochemistry**.** Unstained fibres we defined as Type 2X fibres. The entire muscle section was analysed using a script on imageJ and Jupyter (Python). The variance coefficient was calculated using the minimal fibre Feret's diameter as previously described.^25^

### Mass spectrometry

Approximately 5 ug of gastrocnemius muscle tissue was collected, dissolved in 100 μL lysis buffer (1% sodium deoxycholate, 0.1 M ammoniumbicarbonate), reduced with 5 mM TCEP for 10 min at 95°C, and alkylated with 10 mM chloroacetamide for 30 min at 37°C. Samples were digested with trypsin (Promega, Fitchburg, WI, USA) at 37°C overnight (protein to trypsin ratio: 50:1) and desalted using iST cartridges according to the manufacturer's instructions (Phoenix, PreOmics GmbH, Planegg, Germany).

One microgram of peptides of each sample were subjected to liquid chromatography–mass spectrometry analysis using a dual pressure LTQ‐Orbitrap Elite mass spectrometer connected to an electrospray ion source (both Thermo Fisher Scientific) as recently specified^26^ and a custom‐made column heater set to 60°C. Peptide separation was carried out on an EASY nLC‐1000 system (Thermo Fisher Scientific) equipped with a reversed‐phase high‐performance liquid chromatography column (75 μm × 30 cm) packed in‐house with C18 resin (ReproSil‐Pur C18–AQ, 1.9 μm resin; Dr Maisch GmbH, Ammerbuch‐Entringen, Germany). Separation was achieved by a linear gradient from 95% Solvent A (0.1% formic acid, 99.9% water) and 5% Solvent B (80% acetonitrile, 0.1% formic acid, 19.9% water) to 28% Solvent B over 75 min to 40% Solvent B over 15 min to 95% Solvent B over 2 min and 95% Solvent B over 18 min at a flow rate of 0.2 μL/min.

The data acquisition mode was set to obtain one high resolution mass spectrometry (MS) scan in the FT part of the mass spectrometer at a resolution of 240 000 full width at half maximum (at 400 m/z, MS1) followed by MS/MS (MS2) scans in the linear ion trap of the 20 most intense MS signals. The charged state screening modus was enabled to exclude unassigned and singly charged ions, and the dynamic exclusion duration was set to 30 s. The ion accumulation time was set to 300 ms (MS1) and 50 ms (MS2). MS1 and MS2 scans were acquired at a target setting of 1E6 ions and 10 000 ions, respectively. The collision energy was set to 35%, and one microscan was acquired for each spectrum.

To determine changes in protein expressions across samples, a MS1‐based label‐free quantification was carried out. Therefore, the generated raw files were imported into the Progenesis QI software (Nonlinear Dynamics, Version 2.0) and analysed using the default parameter settings. MS/MS data were exported directly from Progenesis QI in mgf format and searched against a decoy database of the forward and reverse sequences of the predicted proteome from *mus musculus* (Uniprot, download date: 2017/04/18, total of 34,490 entries) using MASCOT (version 2.4.1). The search criteria were set as following: full tryptic specificity was required (cleavage after lysine or arginine residues); 3 missed cleavages were allowed; carbamidomethylation (C) was set as fixed modification and oxidation (M) as variable modification. The mass tolerance was set to 10 ppm for precursor ions and 0.6 Da for fragment ions. Results from the database search were imported into Progenesis QI, and the final peptide measurement list containing the peak areas of all identified peptides, respectively, was exported. This list was further processed and statically analysed using our in‐house developed SafeQuant R script (SafeQuant, https://github.com/eahrne/SafeQuant). The peptide and protein false discovery rate was set to 1% using the number of reverse hits in the dataset.

The result details of the proteomics experiments carried out including identification scores, number of peptides quantified, normalized (by sum of all peak intensities) peak intensities, log2 ratios, coefficients of variations, and *P*‐values for each quantified protein and sample are displayed in Supporting Information, MS data. All raw data and results associated with the manuscript have been deposited in to the ProteomeXchange Consortium *via* the PRIDE^27^ partner repository with the dataset identifier PXD013294 and 10.6019/PXD013294.

### Polysome profiling

One gastrocnemius muscle from each mouse was pulverized under cryogenic conditions in a cryo‐freezer grinder (SpexSamplePrep, 10 cps 3 × 2 min) in 2.5 mL of lysis buffer (20 mM Tris‐HCl, pH = 7, 100 mM NaCl, 50 mM NH_4_Cl, 10 mM MgCl_2_, 1% Triton X‐100) and freshly added 100 μg/mL cycloheximide, 1 mM dithiothreitol supplemented with a protease inhibitor cocktail tablet (Roche), and 200 U of SUPERase*In (Invitrogen). After 2 h incubation at 4°C on a rotating wheel, lysates were disrupted by five up‐and‐down passages through a 19‐gauge syringe and clarified by centrifugation for 10 min at 12 000 rcf at 4°C. Polysome profiling was further carried out as previously described by Ingolia and colleagues.^28^ Briefly, equal loading was assured by measuring OD260 for each sample. Lysates were then layered on top of a pre‐cooled 50–10% sucrose gradient (dissolved in 8.3 mM Tris‐HCl, pH = 7.5, 8.3 mM NH_4_Cl, 2 mM MgCl_2_ freshly supplemented with 0.083 mM dithiothreitol, 100 μg/mL cycloheximide, and 200 U of SUPERase*In). Gradient was formed using a Gradient Master instrument (Biocomp) according to the manufacturer's instruction. Samples were centrifuged at 150 000 rcf for 3 h at 4°C using a Beckman SW41Ti rotor. Profiles of the different samples were obtained at a speed of 0.5 mL/min and continuous measurement of the absorbance at 254 nm.

## Results

### Generation of an inducible, muscle fibre‐specific raptor knockout mouse

**Figure 1:**
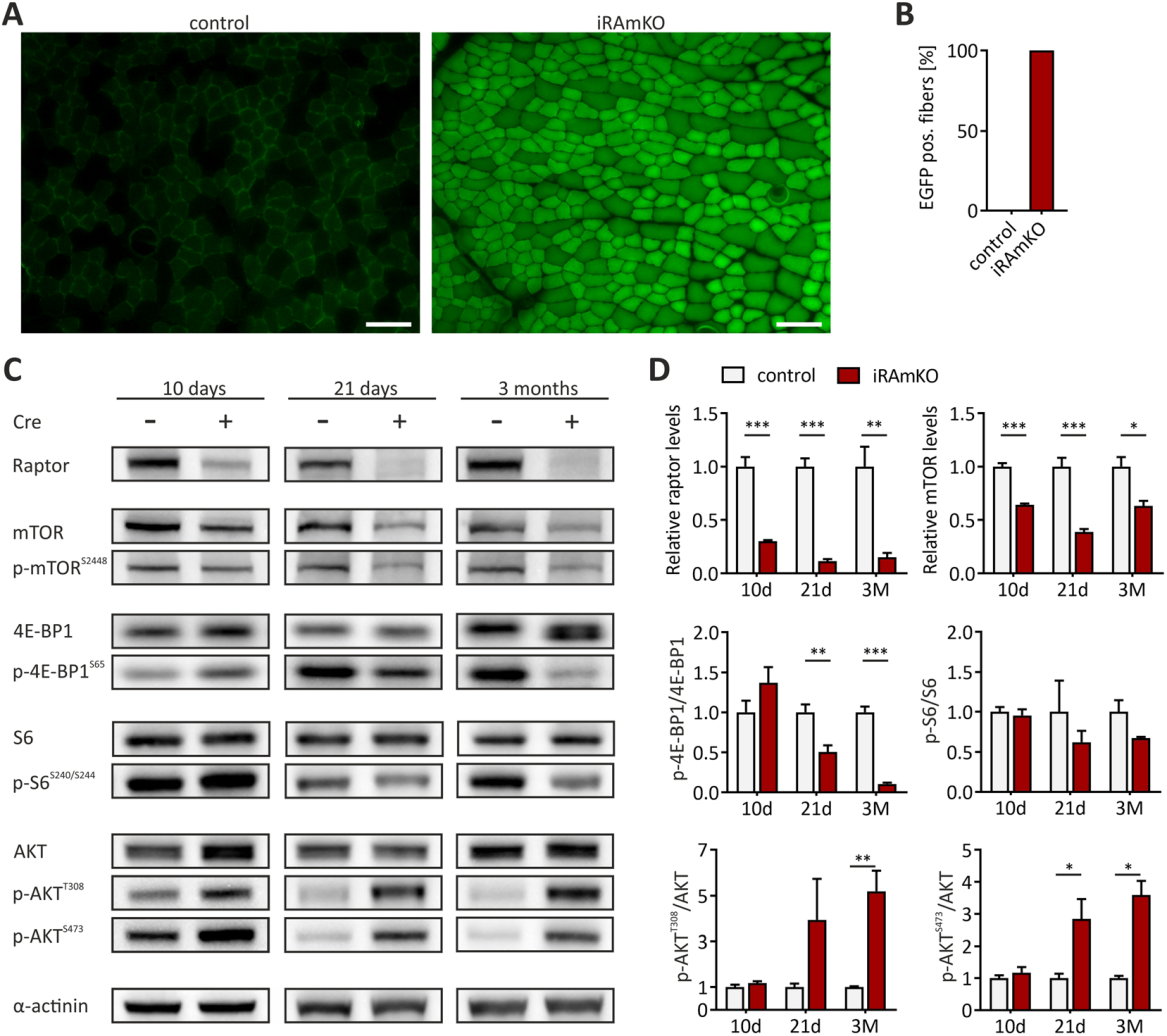
Generation of an inducible, muscle fiber‐specific raptor knockout mouse. (*A*) Tibialis anterior muscle cross‐sections of inducible raptor muscle‐specific knockout 10 days post‐tamoxifen treatment were fixed with paraformaldehyde and imaged for green fluorescent protein. Scale bar = 100 μm. (*B*) Quantification of green fluorescent protein positive fibers. (*C*) Western blot analysis of tibialis anterior lysates from three different time points after tamoxifen injection. Equal amounts of protein were loaded in each lane, and α‐actinin was used as a loading control. Proteins detected in the different immunblots are indicated at the left. (*D*) Quantifications of the immunoblots shown in C. All values were normalized to α‐actinin, and mean values detected in Cre‐negative (control) samples were set to 1. Quantifications for the indicated proteins were done 10 days (10d), 21 days (21d), or 3 months (3M) after tamoxifen injection. 10d and 21d inducible raptor muscle‐specific knockout *n* = 4, 3 month inducible raptor muscle‐specific knockout *n* ≥ 3 (Cre + *n* = 3, Cre−*n* = 4). Values represent the mean ± SEM. Significance was assessed using two‐tailed unpaired student's *t*‐test: *P < 0.05, **P < 0.01, ***P < 0.001. 4E‐BP1, eukaryotic translation initiation factor 4E‐binding protein 1; EGFP, enhanced green fluorescent protein; iRAMKO, inducible raptor muscle‐specific knockout; mTOR, mammalian target of rapamycin complex.

To investigate the role of mTORC1 signalling in adult muscle, we crossed *Rptor* ^fl/fl^ mice^13^ with mice expressing a modified Cre‐recombinase (Mer–Cre–Mer) under the control of the HSA promoter.^20^ We name these mice iRAmKO mice. *Rptor* deletion was induced in 3‐month‐old to 4‐month‐old mice to assure that muscles had reached adult size. Mice were analyzed 10 days, 21 days, 3 months, and 5 months after the induction of *Rptor* deletion. To monitor successful recombination, iRAmKO mice also carried an EGFP‐reporter.^21^ Daily injections of TAM into iRAmKO mice for five consecutive days (Supporting Information, *Figure* *S1* *A*) caused translocation of Mer–Cre–Mer into the myonuclei resulting in recombination of the floxed alleles. The day after the fifth injection was defined as Day 1 of the knockout (Supporting Information, *Figure* *S1* *A*). Ten days after TAM injection, all muscle fibres of the tibialis anterior (TA) were EGFP positive, indicating efficient recombination (*Figure* 1A and 1B; Supporting Information, *Figure* *S1* *B*). To assure complete loss of raptor protein, we also performed a detailed biochemical analysis of the mTOR pathway. Raptor protein levels were ~30% of control after 10 days and ~11% after 21 days, and this low level of raptor remained constant after 3 months (*Figure* 1C and 1D). This residual raptor expression likely originates from non‐targeted cells residing in skeletal muscle as has also been observed in constitutive, muscle fibre‐specific RAmKO mice.^13^ We also examined raptor protein levels in the heart to test tissue specificity. Despite a weak GFP signal, raptor levels were unchanged in the heart (Supporting Information, *Figure* *S1* *B*). In line with previous reports,^8^ loss of raptor protein significantly reduced total levels of mTOR as well as the phosphorylated (Serine2448) levels of mTOR protein (*Figure* 1C and 1D). Phosphorylation of the mTORC1 target 4E‐BP1 was not altered at 10 days post‐injection but was significantly lower 21 days and 3 months after TAM injection (*Figure* 1C and 1D). Levels of p‐S6 showed a similar pattern as p‐4E‐BP1, but they did not reach statistical significance (*Figure* 1C and 1D). The activity of protein kinase B (PKB/AKT) is indirectly controlled via an inhibitory feedback loop involving the mTORC1 target S6 kinase 1.^29^ Accordingly, as previously observed in response to mTORC1 inhibition,^30^ iRAmKO mice display higher AKT phosphorylation at 21 days and 3 months post‐injection at both the PDK1‐dependent (p‐AKT^T308^) and mTORC2‐dependent site (p‐AKT^S473^) (*Figure* 1C and 1D). In conclusion, these experiments show that TAM efficiently depletes raptor in iRAmKO mice leading to abrogation of mTORC1 signalling after 21 days.

### Prolonged depletion of raptor does not cause an overt phenotype

**Figure 2:**
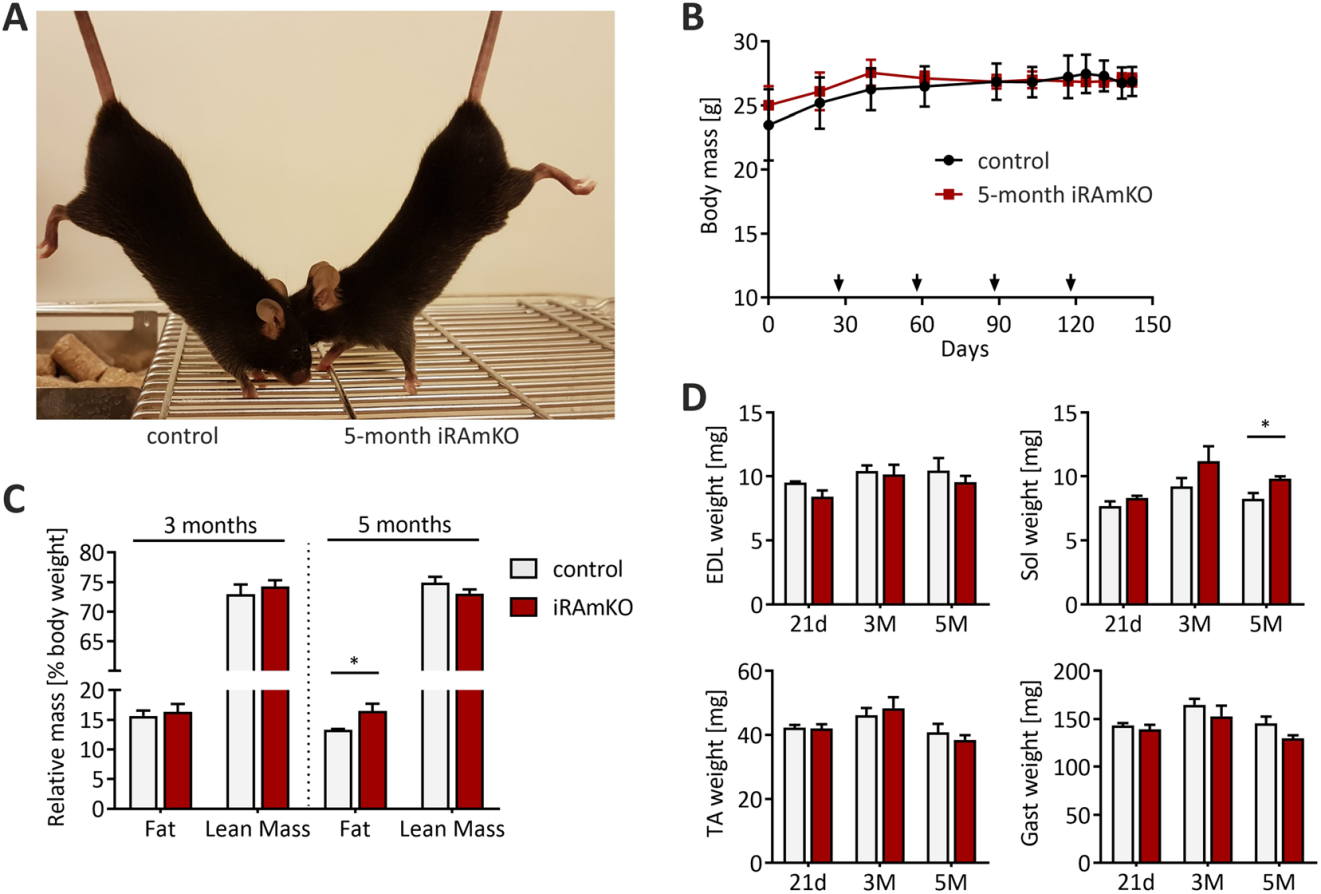
Prolonged depletion of raptor does not cause an overt phenotype. (*A*) Photograph of 8‐month‐old inducible raptor muscle‐specific knockout and control mouse that were 5 month depleted for raptor. (*B*) Weight curve of 5 month inducible raptor muscle‐specific knockout and controls after tamoxifen injection. The arrows indicate the monthly repeated tamoxifen injections. At no time point was there a significant difference in the body mass. (*C*) Relative fat and lean mass measured via MRI normalized to body mass. Three month inducible raptor muscle‐specific knockouts present no changes while 5 month iRAmKOs reveal an increase in fat mass. (*D*) The weight of the four hind limb muscles tibialis anterior, *extensor digitorum longus*, soleus (Sol), and the gastrocnemius (Gast) were measured during dissection in 21 day, 3 months, and 5 month inducible raptor muscle‐specific knockouts. Twenty‐one day and 5 month inducible raptor muscle‐specific knockouts *n* = 4, 3 month inducible raptor muscle‐specific knockouts *n* ≥ 3. Values represent the mean ± SEM. Significance was assessed using two‐tailed unpaired student's *t*‐test: *P < 0.05, **P < 0.01, ***P < 0.001. EDL, *extensor digitorum longus*; iRAMKO, inducible raptor muscle‐specific knockout; TA, tibialis anterior.

To analyse the long‐term consequence of raptor depletion in adult muscle, we treated 3‐month‐old mice with TAM and analysed the mice up to 5 months later. This time point was chosen as developmental, RAmKO mice show a severe myopathy at the age of 5 months and eventually succumb at the age of 5–6 months.^13^ Contrary to what we expected, 5 month iRAmKO mice were barely distinguishable from their control littermates (*Figure* 2A). For example, 5 month iRAmKO mice did not display kyphosis, a sign of muscle weakness. Body mass of mutant mice was not different from littermate controls during the entire period (*Figure* 2B). Similarly, we could not detect any difference in relative fat mass after 3 months and only a slight, but significant, increase of fat mass after 5 months (*Figure* 2C). Lean mass was neither significantly changed at 3 nor at 5 months (*Figure* 2C). Weighing individual muscles showed that *extensor digitorum longus*, soleus, TA, and *gastrocnemius* were not different to the control muscles with the exception of soleus muscle in 5 month iRAmKO mice, which was significantly heavier than the control (*Figure* 2D). Thus, removal of raptor in fully grown muscle for up to 5 months does not promote loss of muscle or body mass.

### Five months of raptor depletion increases fibre size variability but not mean fibre area

**Figure 3:**
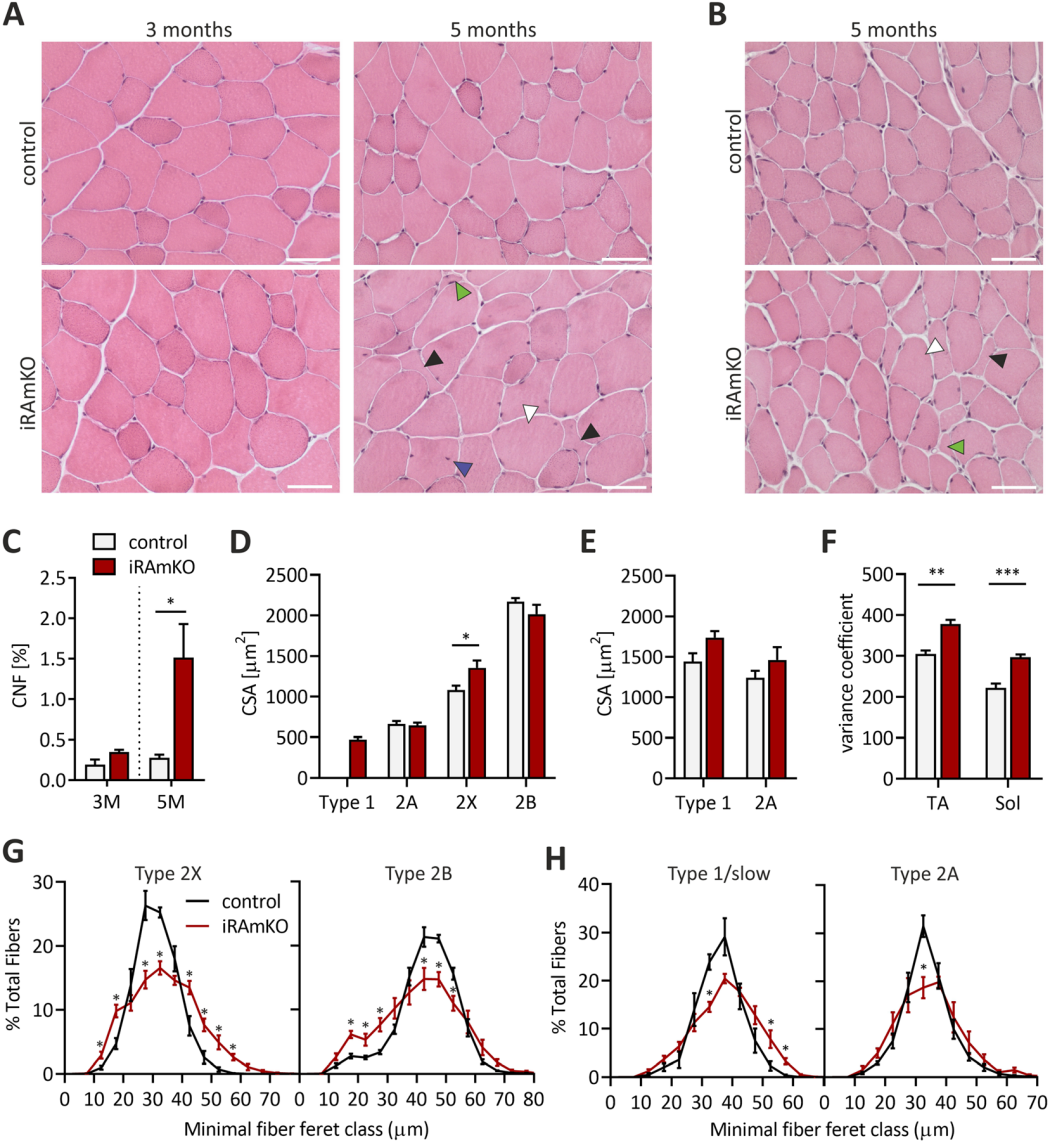
Five months of raptor depletion increases fiber size variability but not mean fibre area. (*A*) Haematoxylin and eosin staining of TA muscle cross‐sections from 3 and 5 month inducible raptor muscle‐specific knockouts and controls. The black arrows indicate fibres with abnormal morphology, green arrows atrophic fibres, white arrows very large fibres, and blue arrows centralized nuclei. (*B*) Haematoxylin and eosin staining of soleus cross‐sections of 5 month iRAmKOs. (*C*) Quantification of central nucleated fibres (CNF) of the entire tibialis anterior cross‐section (using a 4′,6‐diamidino‐2‐phenylindole and antilaminin staining). (*D*) and (*E*) Average cross‐sectional area of individual fibre types from the tibialis anterior (*D*) and soleus (*E*) of 5 month inducible raptor muscle‐specific knockouts. (*F*) Fibre size distribution as the variance coefficient of 5 month inducible raptor muscle‐specific knockouts calculated using the minimal feret's diameter method.^25^ (*G*) Fibre size distribution of Type 2X and 2B fibres in the tibialis anterior of 5 month inducible raptor muscle‐specific knockouts and controls. (*H*) Fibre size distribution of Type 1/slow and 2A fibres in the soleus of 5 month inducible raptor muscle‐specific knockouts and controls. Three month inducible raptor muscle‐specific knockouts *n* ≥ 3 and 5 month inducible raptor muscle‐specific knockouts *n* = 4. Values represent the mean ± SEM. Significance was assessed using two‐tailed unpaired student's *t*‐test: *P < 0.05, **P < 0.01, ***P < 0.001 (for *G* and *H* only *P was used). Scale bars = 50 μm. CSA, cross‐sectional area; iRAMKO, inducible raptor muscle‐specific knockout.

Both RAmKO and skeletal muscle fibre‐specific mTOR knockout mice (mTORmKO) suffer from a severe, lethal myopathy,^13^, ^14^ which is very pronounced at 6 weeks^14^ and 3 months^13^ of age, respectively. The RAmKO and mTORmKO myopathy is characterized by smaller fibre sizes, increased fibre size variation, centralized myonuclei, central core‐like structures, fibrosis, and inflammation. To examine whether iRAmKO mice display similar alterations, we examined cross‐sections from TA muscles 21 days, 3 months, and 5 months after TAM injection. Twenty‐one day iRAmKOs depicted no changes (Supporting Information, *Figure* *S2* *A*), consistent with a previous report.^8^ After 3 months, despite some fibres from iRAmKO mice appearing more roundish (*Figure* 3A) and an increased variation in Type 2A fibre size distribution and variance coefficient^25^ (Supporting Information, *Figure* S2B and S2D), overall fibre size, fibre size distribution, and variance coefficient were unchanged compared with control littermates (Supporting Information, *Figure S2C, S2E*, *S2F,* and *S2G*). Furthermore, we did not detect an increase in the number of central‐nucleated fibres (*Figure* 3C).

The first myopathic features were observable in H&E stainings of the TA muscle from 5 month iRAmKOs. These included higher numbers of very small and large fibres, centrally nucleated fibres, and a loss of typical polygonal fibre shape (*Figure* 3A and 3C). Similar changes were also observed in soleus muscle cross‐sections from 5 month iRAmKOs (*Figure* 3B). Mean fibre‐type‐specific and overall fibre area and minimal feret diameter were unchanged in 5 month iRAmKOs in both the TA and soleus, with the exception of Type 2X fibres in the TA, which were actually hypertrophic (*Figure* 3D, 3E, and Supporting Information, *Figure* *S2* *H,* *S2* *I*). On the other hand, there were high numbers of both small and large fibres in TA and soleus, as evidenced by a significantly higher variance coefficient^25^ (*Figure* 3F, Supporting Information, *Figure* *S2* *J*, *S2* *K*). The fibre size distribution of individual fibre types similarly did not show a clear shift of the mode but presented a lowering of the most abundant fibre size (*Figures* 3G and 3H; Supporting Information, *Figure* *S2* *L*). This broadening of the fibre size distribution in iRAmKO mice agrees with the increase in the variance coefficient (*Figure* 3F). In conclusion, raptor depletion in fully grown muscle does not induce a generalized muscle atrophy, as observed in RAmKOs^13^ and mTORmKO^14^ but leads to greater heterogeneity in fibre size. Our data also show that it takes 5 months to detect signs of a myopathy.

### Inducible raptor muscle‐specific knockout mice have a reduced oxidative capacity and shift the fibre‐type composition towards slower fibre types

**Figure 4:**
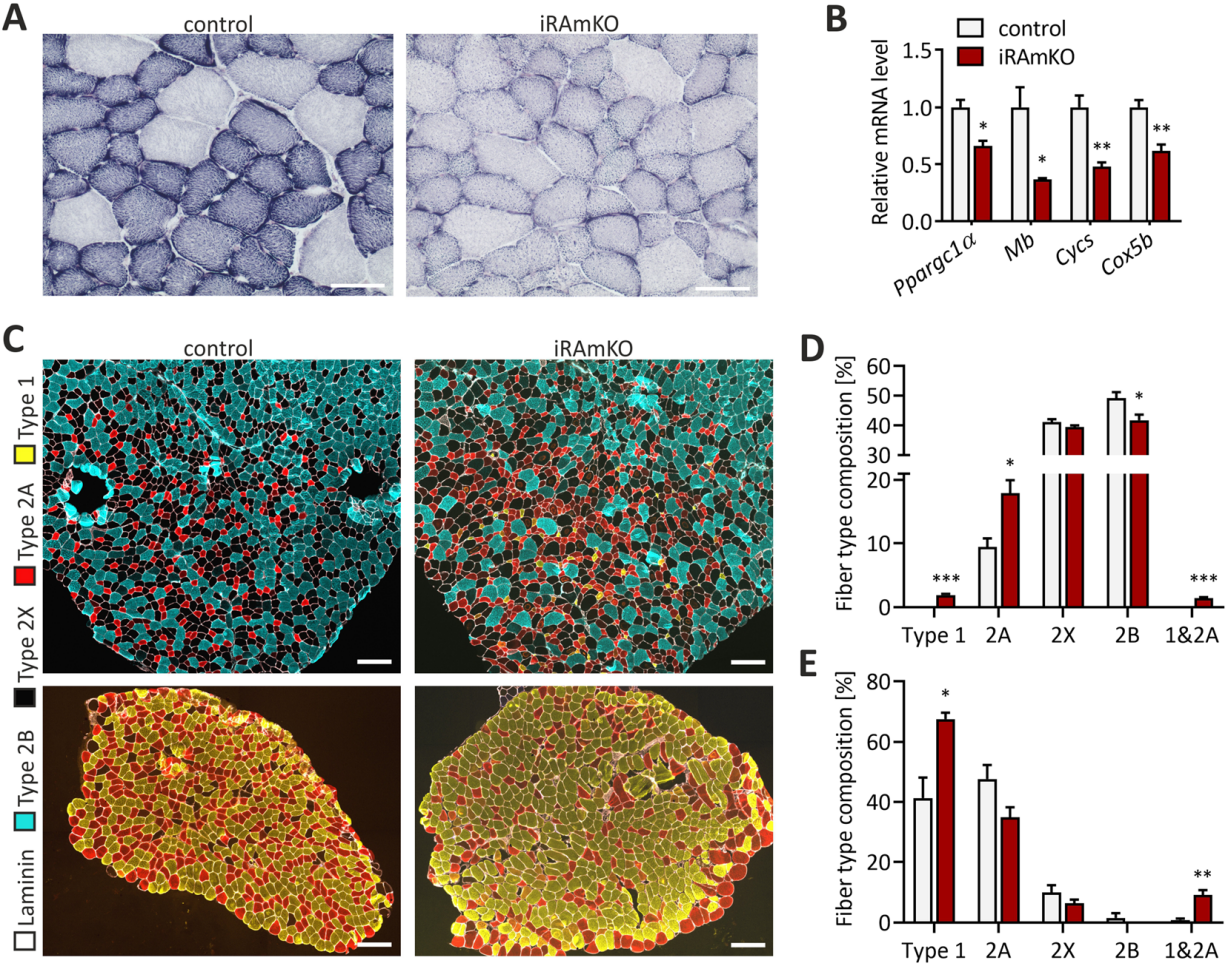
Inducible raptor muscle‐specific knockout mice have a reduced oxidative capacity and shift the fibre‐type composition towards slower fibre types. (*A*) The activity of oxidative enzymes was examined by nicotinamide adenine dinucleotide‐tetrazolium staining on tibialis anterior cross‐sections of 3 month inducible raptor muscle‐specific knockouts. Scale bar = 50 μm. (*B*) Relative mRNA levels of *Ppargc1α* (PGC1α), *Mb* (myoglobin), *Cycs* (cytochrome c), and *Cox5b* (cytochrome c oxidase subunit 5B) in the soleus muscle of 3 month inducible raptor muscle‐specific knockouts. (*C*) Immunohistochemical (IHC) staining against laminin (white), MyHC Type 2B (cyan), MyHC Type 2A (red), MyHC Type 1/slow (yellow), and Type 2X (unstained). The top image is the tibialis anterior, and the lower image is the soleus of a 5 month inducible raptor muscle‐specific knockout and control. Scale bar = 200 μm. (*D*) and (*E*) Quantification of the fibre type composition in tibialis anterior (*D*) and soleus (*E*) of 5 month inducible raptor muscle‐specific knockouts and their control littermates. *n* ≥ 3 for 3 month inducible raptor muscle‐specific knockouts and *n* = 4 for 5 month inducible raptor muscle‐specific knockouts. Values represent the mean ± SEM. Significance was assessed using two‐tailed unpaired student's *t*‐test: *P < 0.05, **P < 0.01, ***P < 0.001. iRAMKO, inducible raptor muscle‐specific knockout.

Another striking phenotype of RAmKO and mTORmKO mice is the significant loss of oxidative capacity, the increase in the number of glycolytic fibres, and the change to more slow‐type contraction properties.^13^, ^14^ In both mouse models, loss of oxidative capacity is based on the downregulation of peroxisome proliferator‐activated receptor gamma coactivator 1‐α (PGC1α), as transgenic overexpression of PGC1α and pharmacological activation of mitochondrial biogenesis is sufficient to normalize oxidative capacity.^31^ To assess the metabolic status of iRAmKO mice, we first performed a nicotinamide adenine dinucleotide‐tetrazolium (NADH‐TR) staining on TA cross‐sections. Although mTORC1 signalling was largely abrogated 21 days after TAM injection, the oxidative properties of iRAmKO mice were not different to controls (Supporting Information, *Figure* *S3* *A*). In contrast, 3 months after TAM injection, iRAmKO mice showed a strong decrease in NADH‐TR staining (*Figure* 4A). In line with the NADH‐TR staining, gene expression of the transcriptional co‐activator PGC1α (*Ppargc1a*) and some of its effector genes, such as myoglobin (*Mb*), cytochrome c (*Cycs*), and cytochrome c oxidase subunit 5B (*Cox5b*), were not altered in 21 day iRAmKOs (Supporting Information, *Figure* *S3* *B*) but were significantly reduced after 3 months (*Figure* 4B).

Fibre‐type composition of skeletal muscle is determined by cell intrinsic and extrinsic mechanisms. Muscle fibres formed in the initial wave of myogenesis express largely developmental and slow forms of myosin heavy chains (MyHCs), while muscle fibres formed postnatally express fast MyHCs.^32^ In addition, the pattern of slow and fast MyHCs in the adult is largely determined by motor innervation. Thus, the observed shift in fibre‐type composition in RAmKO mice might be because of changes in the intrinsic properties in muscle fibres formed during myogenesis. If this hypothesis were correct, depletion of raptor in adult muscle should not affect fibre‐type composition. To test this, we stained muscle cross‐sections with antibodies against MyHCs specific for different fibre types. The TA of both 3 and 5 month iRAmKOs presented a two‐fold increase of Type 2A fibres and a significant reduction of Type 2B fibres (*Figure* 4C and 4D; Supporting Information, *Figure S3C*). Furthermore, while Type 1 fibres are absent in the TA of control mice, ~2% of fibres in 5 month iRAmKOs are positive for Type 1 (*Figure* 4C and 4D). In line with a fast‐to‐slow fibre type transition, most Type 1 fibres are also positive for 2A fibre‐type staining (*Figure* 4D). Soleus muscle, which contains ~40% Type‐1‐positive fibres in control mice, contained more than 60% MyHC‐1‐positive fibres in 5 month iRAmKO mice (*Figure* 4C and 4E). As in the TA, soleus muscle also exhibited a significant number of Type 1 and Type‐2A‐double‐positive fibres (*Figure* 4E). This observation is also confirmed by the MS analysis of the gastrocnemius 3 months post‐TAM treatment, which showed an increased presence of other proteins and isoforms typically expressed in Type 2A and/or slow fibre types, such as Myomesin‐3,^33^ Myosin light chain 6B, Myosin light chain 3, and slow skeletal muscle troponin T (Supporting Information, MS data). Thus, as in RAmKO mice, knockout of raptor in fully grown muscle causes a reduced oxidative capacity and triggers a switch towards slower fibre types.

### Muscles of inducible raptor muscle‐specific knockout mice present slow contractile properties and similar force values to controls

**Figure 5:**
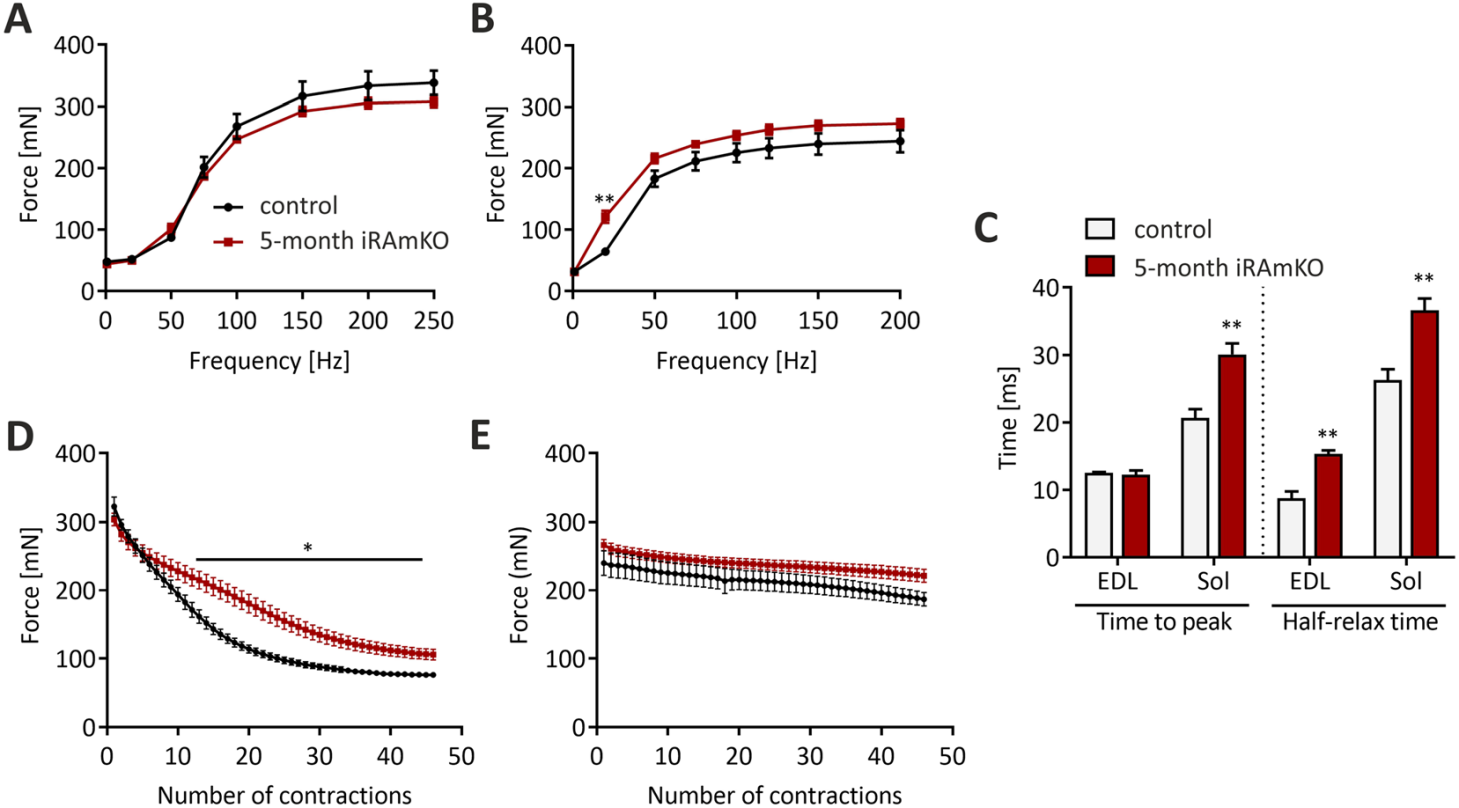
Muscles of inducible raptor muscle‐specific knockout mice present slow contractile properties and similar force values to controls. (*A*) and (*B*) *Ex vivo* muscle force of the *extensor digitorum longus* (*A*) and the soleus (*B*) of 5 month inducible raptor muscle‐specific knockout and control mice was measured at different frequencies. (*C*) Time‐to‐peak and half‐relaxation time of single twitch contractions of *extensor digitorum longus* and soleus muscles in 5 month inducible raptor muscle‐specific knockout and control mice. Note the significant prolongation of time components in inducible raptor muscle‐specific knockout mice because of the shift to slower fibre types. (*D*) *Ex vivo* fatigue resistance measured during 6 min with a 200 Hz stimulus every 8 s. The absolute values are illustrated to show that the muscle force is not decreased. (*E*) The same fatigue protocol measured in soleus muscle at 120 Hz. 5 month inducible raptor muscle‐specific knockout *n* = 4. Values represent the mean ± SEM. Significance was assessed using two‐tailed unpaired student's *t*‐test: *P < 0.05, **P < 0.01, ***P < 0.001. iRAMKO, inducible raptor muscle‐specific knockout.

To test how muscle function is affected by long‐term mTORC1 inactivation, we performed *ex‐vivo* muscle force measurements. The absolute tetanic force of EDL and soleus was not significantly reduced in 5 month iRAmKO mice compared with controls (*Figure* 5A and 5B). Specific force (i.e. force normalized to muscle cross‐sectional area) in response to stimulation frequency was also very similar in iRAmKO and control mice (Supporting Information, *Figure S4A* and *S4B*). Force elicited by a single twitch at 1 Hz was not significantly different in EDL or soleus muscle of iRAmKO compared with controls (*Figure* 5A and 5B). However, the time‐to‐peak twitch force and half‐relaxation time were significantly increased in the soleus muscle of iRAmKO mice compared with littermate controls (*Figure* 5C). In EDL, we could not detect any difference in the time‐to‐peak but half‐relaxation time was significantly increased in iRAmKOs (*Figure* 5C). Importantly, the EDL muscle of iRAmKO mice was significantly more fatigue‐resistant than control EDL (*Figure* 5D). Our fatigue protocol did not reveal a further increase in fatigue resistance of the iRAmKO soleus muscle (*Figure* 5E). Together, these data show that depletion of raptor in fully grown, adult muscle for 5 months does not lead to a significant reduction in force but does affect muscle contraction properties in line with the fast‐to‐slow transition in fibre‐type composition. We thus conclude that the loss of muscle force in RAmKO^13^ and mTORmKO^14^ mice is likely a consequence of impaired growth, whereas the shift in fibre‐type composition may rather be because of mTORC1‐driven changes in muscle homeostasis.

### Removal of mammalian target of rapamycin complex 1 leads to a reduction in translational components

**Figure 6:**
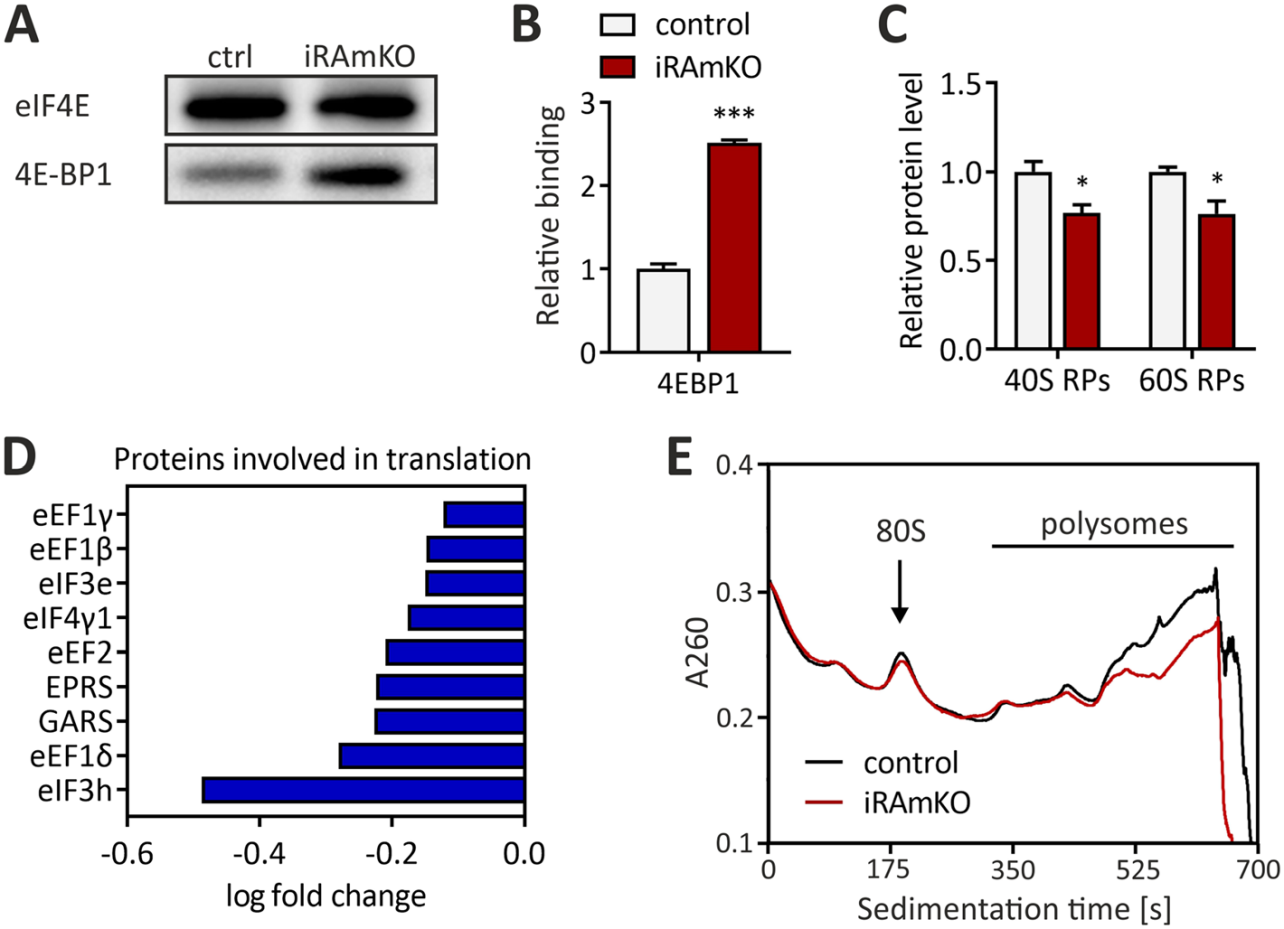
Removal of mammalian target of rapamycin complex 1 leads to a reduction in translational components**.** (*A*) Western blot of an 7‐methylguanosine 5′‐triphosphate pulldown on gastrocnemius lysates of 3 month inducible raptor muscle‐specific knockouts with the primary antibodies against eukaryotic translation initiation factor 4E and eukaryotic translation initiation factor 4E‐binding protein 1. (*B*) Quantification of the 7‐methylguanosine 5′‐triphosphate pulldown. The levels of eukaryotic translation initiation factor 4E‐binding protein 1 were normalized to the amount of eukaryotic translation initiation factor 4E. (*C*) Average relative expression levels of the ribosomal proteins obtained from the mass spectrometry data, separated into 40S and 60S subunits. (*D*) Significantly decreased proteins involved in translation from the mass spectrometry data (excluding false discovery rate). (*E*) Average polysome profiles of gastrocnemius from 3 month inducible raptor muscle‐specific knockouts and controls. The monosomes are labelled as 80S. Three month inducible raptor muscle‐specific knockouts and controls *n* = 3. Values represent the mean ± SEM. Significance was assessed using two‐tailed unpaired student's *t*‐test: *P < 0.05, **P < 0.01, ***P < 0.001. 4E‐BP1, eukaryotic translation initiation factor 4E‐binding protein 1; eIF4E, eukaryotic translation initiation factor 4E, iRAMKO, inducible raptor muscle‐specific knockouts.

Mammalian target of rapamycin complex 1 controls translation predominantly through phosphorylation of 4E‐BP1 and the subsequent release of eIF4E.^7^ In iRAmKO mice, inhibition of 4E‐BP1, as indicated by its phosphorylation, is strongly reduced (*Figure* 1C and 1D). We examined whether this also led to greater binding of 4E‐BP1 to eIF4E by an m7GTP‐pulldown followed by western blot analysis. The ratio of 4E‐BP1 to eIF4E was two‐point‐five‐fold higher in iRAmKO mice compared with control, indicating increased inhibition of translation initiation (*Figure* 6A and 6B). Transcripts that contain a 5′ terminal oligopyrimidine (TOP) or a TOP‐like tract show the highest reduction in translation upon 4E‐BP1 inhibition.^7^ To test whether this was also the case in skeletal muscle, we used MS to quantify the mean level of 40S and 60S ribosomal proteins, which mostly derive from TOP mRNA. 7, 34 Indeed, levels of ribosomal proteins were reduced by ~24% in mice deficient for raptor (*Figure* 6C). Similarly, several translation initiation and elongation factors as well as some aminoacyl‐tRNA synthetases were strongly reduced (*Figure* 6D). Together, these data suggest an overall suppression of protein translation in skeletal muscle of iRAmKO mice. To further test this, we performed polysome profiling on the gastrocnemius muscle of 3 month iRAmKOs. Typically, a decrease in the translation rate lowers the number of polysomes and increases the number of monosomes and of 40S and 60S ribosomal subunits.^7^, ^35^ Indeed, we detected a decrease in the signal from high polysomes in the iRAmKOs (*Figure* 6E). Neither an increase in the monosome peak nor an increase in 40S and 60S subunits was visible. This lack of an increase of non‐polysome ribosomal subunits in iRAmKO muscle is possibly because of the decrease in ribosomal proteins. Hence, removal of raptor does lead to a reduction in global translation, consistent with the results of others.^8^ Despite this reduction, iRAmKO muscles do not display any atrophy up to 5 month post‐raptor depletion (*Figures* 2D and 3).

## Discussion

Using experimental paradigms of hypertrophy, it has been clearly demonstrated that mTORC1 inhibition blunts hypertrophy.^8^, ^9^, ^10^ However, definitive evidence for a role of mTORC1 in the maintenance of adult muscle mass is largely missing. To address this question, we established a mouse model in which we deleted *Rptor* in adult skeletal muscle. Careful characterization of this inducible muscle knockout showed that (i) all muscle fibres undergo Cre‐mediated deletion as indicated by the GFP‐positivity and (ii) 21 days after TAM injection is sufficient to abrogate mTORC1 signalling. Strong depletion of raptor protein preceded complete inhibition of mTORC1 signalling, as the amount of raptor was already reduced by 70% after 10 days of TAM treatment without any reduction in 4E‐BP1 phosphorylation. These data suggest that mTORC1 has quite a long half‐live and that low levels of the protein complex are sufficient to maintain normal signalling. Consistent with these data, others have also observed that raptor levels continue to decrease until Day 14 after *Rptor* deletion.^8^ Concomitant with the depletion of raptor, levels of mTOR drop significantly, indicating, as has been suggested from the mTORC1 structure,^36^ an important role of raptor for the stability/folding of mTOR *in vivo*.

Constitutive, muscle‐specific knockout of raptor or mTOR causes a severe, early‐onset myopathy that eventually causes the death of the mice. In those mice, body and muscle mass is significantly smaller at the age of 6 to 12 weeks. At the age of 5 months, close to 50% of the RAmKO or mTORmKO mice have died because of the severe myopathy.^13^, ^14^ Hence, the most striking result of our current study is the very mild phenotype and the lack of any muscle loss in iRAmKO mice during the 5 month observation period. In 3 month iRAmKO mice, spinal curvature, body, and muscle mass were not different from wild‐type controls. Furthermore, histological analysis of TA muscle did not show any myopathic signs, such as centralized nuclei, mononuclear cell infiltration, or fibrosis, and overall, the variance coefficient was not significantly different. Even after 5 months of raptor depletion, mice still did not show any overt myopathic signs, such as kyphosis, muscle mass loss, or muscle weakness. At this late time point, only early signs of a myopathy started to become visible in muscle histology such as increased fibre size variation and ~1.5% of central nucleated fibres. These results indicate that the early‐onset myopathy observed in RAmKO and mTORmKO mice might be a consequence of impaired muscle hyperplasia or the requirement of juvenile muscle to grow. Moreover, muscle histology shows very mild myopathic signs in 5 month iRAmKO mice that were never as severe as the myopathy described for 3‐month‐old or 5‐month‐old RAmKO mice.^13^ We are aware that this experiment does have limitations, in particular because it takes approximately three weeks to eliminate mTORC1 signalling completely. Nevertheless, it provides additional evidence that mTORC1 signalling is not highly critical for skeletal muscle maintenance under sedentary, physiological conditions. Our results are thus different from those obtained when the increase in load causes muscle hypertrophy. In all those paradigms, mTORC1 signalling strongly contributes to hypertrophy.^8^, ^9^, ^10^

The main role of mTORC1 is to control cell growth by affecting protein synthesis.^37^ As protein synthesis is important in the S‐phase of mitosis, mTORC1 dysfunction strongly affects, although indirectly, fast‐dividing cells, including myogenic precursors.^15^ Skeletal muscle is a post‐mitotic, multinucleated tissue, which does not alter cell size fundamentally unless challenged. To assess whether raptor depletion would also affect protein translation, we thus conducted proteomics in gastrocnemius muscle from 3 month iRAmKO mice and m7GTP pulldown experiments. Consistent with results obtained in mouse embryonic fibroblasts,^7^ ribosomal proteins were strongly and significantly reduced in 3 month iRAmKO mice. The significant loss of ribosomes was accompanied with a decrease in several initiation and elongation factors, such as eIF3, eIF4, and eEF1. Interestingly, all of these downregulated proteins are encoded by TOP mRNAs.^34^ Consistent with a reduction in overall protein synthesis, we observed a relative loss of polysomes in the lysates of iRAmKO muscle. Thus, our results show that TOP mRNAs are also the main target of the translational activity of mTORC1 in skeletal muscle as in other cells,^7^, ^8^ including cancer cells.^38^ It is thus even more surprising that several months of mTORC1 inactivity does not cause any detectable loss of muscle mass. We hypothesize that a combination of several reasons could explain this lack of phenotype. First, the lowering of TOP mRNA translation may be compensated for by increased translation of other mTORC1‐independent transcripts. Indeed, iRAmKO muscles contain increased levels of several proteins (for example several proteins characteristic of slow muscle fibres; see Supporting Information, MS data). Secondly, the major components of the muscle, for example myosin and titin, that together constitute ~34% of muscle mass,^39^ may not be translated cap‐dependently, and there may not be a great need to synthesize them at high rates because of their low turnover rate under basal conditions.^40^, ^41^

While muscle size and myopathic features greatly differed between iRAmKO and RAmKO mice, changes in the metabolism and the fibre‐type composition were comparable. A particularly interesting observation is the shift towards slower fibre types. It was previously shown that RAmKOs have a higher proportion of Type 1 fibres; however, it was unclear whether this was a consequence of developmental effects.^13^ Myofibres positive for slow MyHC can still be detected up to 4 weeks after birth in fast muscles, which then vanish later in development.^42^, ^43^ Hence, one possibility was that raptor deficiency would prevent this shift to fast type muscle fibres. Our data now show that loss of mTORC1 signalling is sufficient to induce a shift towards slower fibre isoforms in the adult. As raptor is ablated only in muscle, this shift is likely based on muscle‐intrinsic, raptor‐dependent processes. Interestingly, mTORmKO mice also show this fibre‐type shift,^14^ indicating that this phenotype is a result of reduced mTORC1 signalling rather than a raptor‐specific effect. Two well‐described transcription factors involved in the slow fibre switch program are NFATc1 and MEF2.^44^, ^45^, ^46^ Levels of MEF2A and MEF2D are both increased in RAmKO muscle,^13^ and NFATc1 has been shown to be negatively regulated by mTORC1.^47^ Yet, in both cases, it is unclear whether mTORC1 directly affects these proteins. For example, studies have suggested that the slow removal of calcium from the sarcoplasm characteristic of slow muscle fibres activates both NFATc1 and MEF2.^13^ Hence, changes in calcium handling in raptor‐depleted muscle may underlie the fibre‐type switch. However, the increased removal time of calcium may also be simply a consequence and not a cause of the fast to slow switch. Another protein that may be involved and shows increased activity in raptor‐depleted muscle is AKT. Activated AKT inhibits GSK3β, which promotes NFATc1 nuclear entry.^48^ High AKT activity can also lead to increased MEF2 expression.^49^ Thus, constitutively active AKT in iRAmKOs may contribute to the fibre switch. Indeed, time‐to‐peak and half relaxation time increase in muscles that express a constitutively active form of AKT.^50^

In conclusion, we generated an inducible RAmKO mouse model that allows the analysis of mTORC1 inhibition in fully grown muscle. We show that raptor knockout in adult muscle has a much weaker effect than expected. Despite a loss of mTORC1 signalling and a significant loss of ribosomes and protein synthesis, muscle mass at no stage becomes significantly less than controls. However, similar to RAmKO and mTORmKO mice, the fast‐to‐slow fibre‐type switch and reduction in oxidative capacity is already visible after 3 months. Thus, this mouse model may allow a better understanding of the mechanisms involved in the antiageing activity of the allosteric mTORC1 inhibitor rapamycin.^13^, ^14^

## Funding

This study was funded by the Cantons of Basel‐Stadt and Basel‐Landschaft and by a grant from the Swiss National Science Foundation.

## Conflict of interest

The authors declare no conflicts of interest.

## Author Contributions

ASH designed and performed experiments, analysed the data, and wrote the manuscript. KC generated the mouse line and helped in the initial phase of the project, LAT performed the polysome profiling, SL performed the muscle force measurements, AS performed the proteomics study, DJH helped analyse the data and discussed the project, MS provided some funding for the project, and MAR conceived the project, secured funding, analysed and discussed data, and wrote the manuscript.

## Supporting information


**Figure S1.** (A) Timeline of TAM injections. (B) Western blot analysis of heart and TA lysates using antibodies directed against the proteins indicated. Note that despite low levels of EGFP expressed in the heart, raptor levels were unchanged, suggesting minimal expression of Cre in this tissue. EGFP was highly expressed in the tibialis anterior (TA) muscle and raptor was strongly reduced.
**Figure S2.** (A) H&E staining of TA cross‐sections from 21‐day iRAmKOs depicted no changes. Scale bar = 50 μm. (B) The variance coefficient using the minimal feret diameter showed an increase in fibre size distribution for type 2A fibers in the TA of 3‐months iRAmKOs. (C) All fiber types of the TA together, however presented no changes in the variance coefficient. (D) Fiber size distribution of type 2A, 2X and 2B fibers from the TA of 3‐months iRAmKOs. (E) Average cross‐sectional area (CSA) of all fibers and (F) average CSA of individual fiber types from the TA of 3‐month iRAmKOs G) average minimal fiber feret diameter (f. f. d.) of all fibers from the TA of 3‐month iRAmKOs and their control littermates. (H) Average CSA and (I) average minimal f. f. d. of all fibers in the TA and soleus (Sol) of 5‐month iRAmKOs. (J) Variance coefficient of individual fiber types in the TA and (K) soleus. (L) Fiber size distribution of type 2A fibers of the TA from 5‐month iRAmKOs. n ≥ 3 (3M) and n = 4 (5M). Values represent the mean ± SEM. Significance was assessed using two‐tailed unpaired student's t‐test: *p < 0.05, **p < 0.01, ***p < 0.001.
**Figure S3.** (A) NADH‐TR staining of TA cross‐sections from 21‐day iRAmKOs. (B) Relative mRNA levels of *Ppargc1α* (PGC1α), *Mb* (myoglobin), *Cycs* (cytochrome c) and *Cox5b* (cytochrome c oxidase subunit 5B) in the *soleus* muscle of 21‐day iRAmKOs. *C*) Fiber type composition of the TA from 3‐month iRAmKOs. n = 4 for 21d iRAmKOs and n ≥ 3 for 3M iRAmKOs. Values represent the mean ± SEM. Significance was assessed using two‐tailed unpaired student's *t*‐test: *p < 0.05, **p < 0.01, ***p < 0.001.
**Figure S4.** (A) and (B) Specific *ex vivo* muscle force (normalised to the muscle size as explained in material and methods) of the EDL (A) and the *soleus* (B) of 5‐month iRAmKOs and controls. 5‐month iRAmKOs and controls n = 4. Values represent the mean ± SEM. Significance was assessed using two‐tailed unpaired student's *t*‐test: *p < 0.05, **p < 0.01, ***p < 0.001.
**Table S1.** All quantifications of the western blots shown in Fig. 1C. All values were normalised on α‐actinin and on control samples. 10 and 21‐day iRAmKOs n = 4, 3‐month iRAmKOs n ≥ 3. Values represent the mean ± SD. Significance was assessed using two‐tailed unpaired student's *t*‐test: *p < 0.05, **p < 0.01, ***p < 0.001.
**Table S2.** All quantifications of the western blots shown in Figure S1B. All values were normalised on α‐ actinin and on control samples. 3‐month iRAmKOs n ≥ 3. Values represent the mean ± SD. Significance was assessed using two‐tailed unpaired student's t‐test: *p < 0.05, **p < 0.01, ***p < 0.001.

**Data S1.** Supporting Information
