# Supplementary material for: mTORC1 signalling is not essential for the maintenance of muscle mass and function in adult sedentary mice
Source: J Cachexia Sarcopenia Muscle. 2019 Nov 7;11(1):259–73. doi: 10.1002/jcsm.12505 (PMC7015237; doi:10.1002/jcsm.12505)

Figure S1

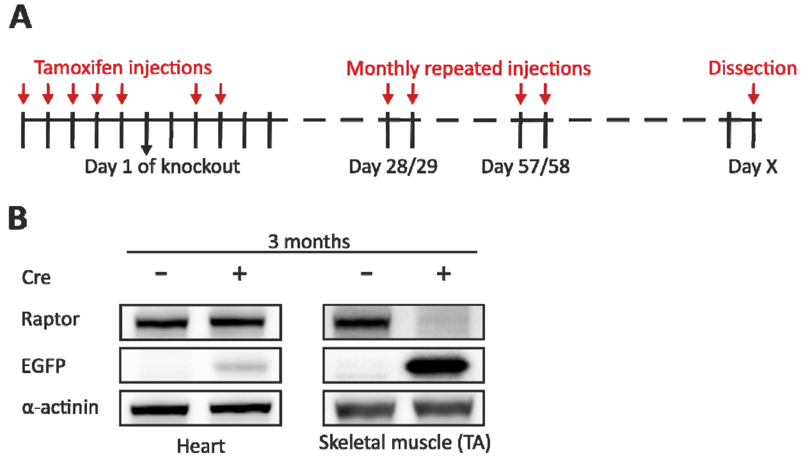

Figure S1. (A) Timeline of TAM injections. (B) Western blot analysis of heart and TA lysates using antibodies directed against the proteins indicated. Note that despite low levels of EGFP expressed in the heart, raptor levels were unchanged, suggesting minimal expression of Cre in this tissue. EGFP was highly expressed in the *tibialis anterior* (TA) muscle and raptor was strongly reduced.

Figure S2

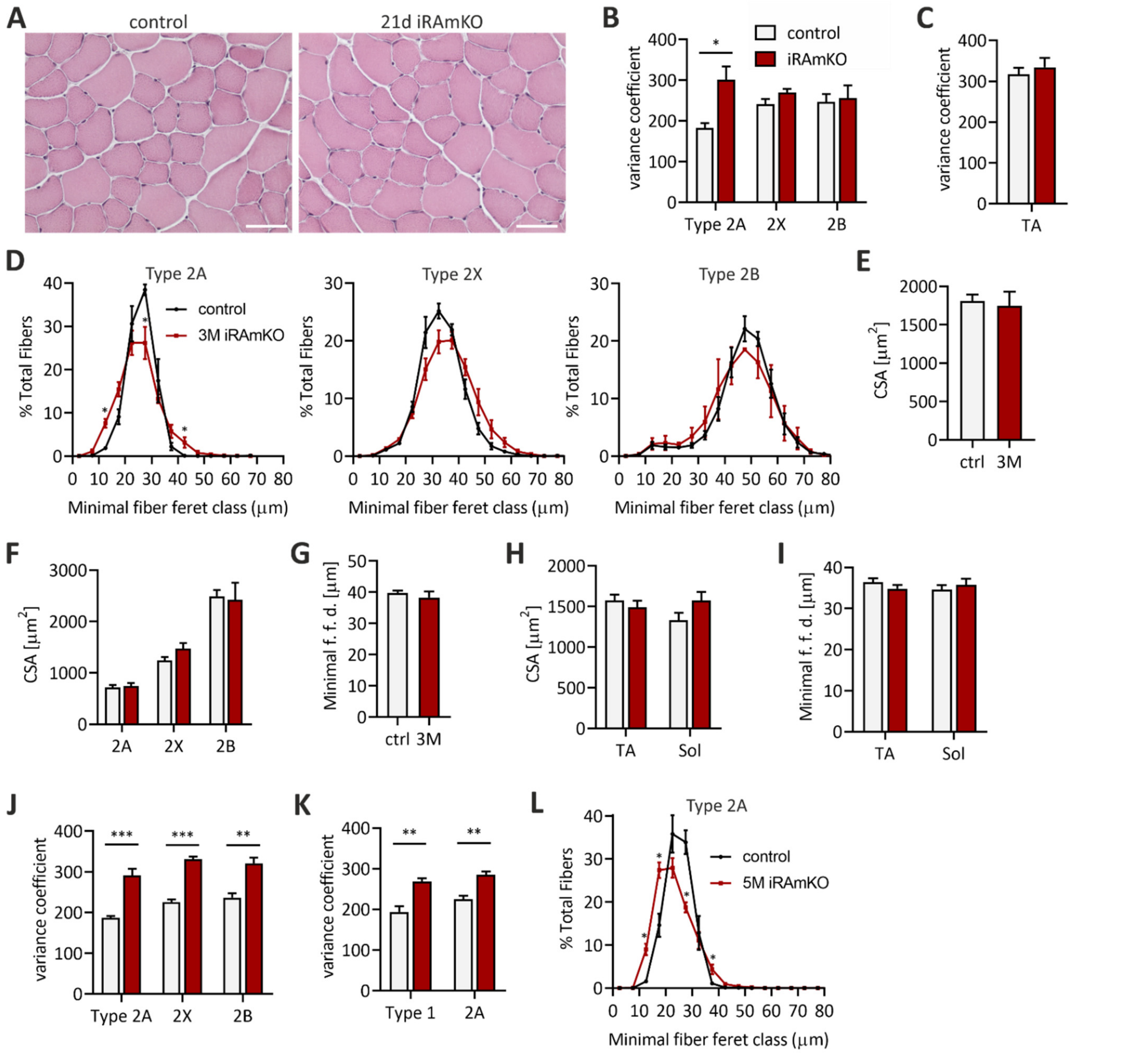

Figure S2. (A) H&E staining of TA cross-sections from 21-day iRAmKOs depicted no changes. Scale bar = 50 µm. (B) The variance coefficient using the minimal feret diameter showed an increase in fiber size distribution for type 2A fibers in the TA of 3-months iRAmKOs. (C) All fiber types of the TA together, however presented no changes in the variance coefficient. (D) Fiber size distribution of type 2A, 2X and 2B fibers from the TA of 3-months iRAmKOs. (E) Average cross-sectional area (CSA) of all fibers and (F) average CSA of individual fiber types from the TA of 3-month iRAmKOs *G*) average minimal fiber feret diameter (f. f. d.) of all fibers from the TA of 3-month iRAmKOs and their control littermates. (H) Average CSA and (I) average minimal f. f. d. of all fibers in the TA and *soleus* (Sol) of 5-month iRAmKOs. (J) Variance coefficient of individual fiber types in the TA and (K) *soleus*. (L) Fiber size distribution of type 2A fibers of the TA from 5-month iRAmKOs. $n \geq 3$ (3M) and $n = 4$ (5M). Values represent the mean ± SEM. Significance was assessed using two-tailed unpaired student's *t*-test: *$p < 0.05$, **$p < 0.01$, ***$p < 0.001$.

Figure S3

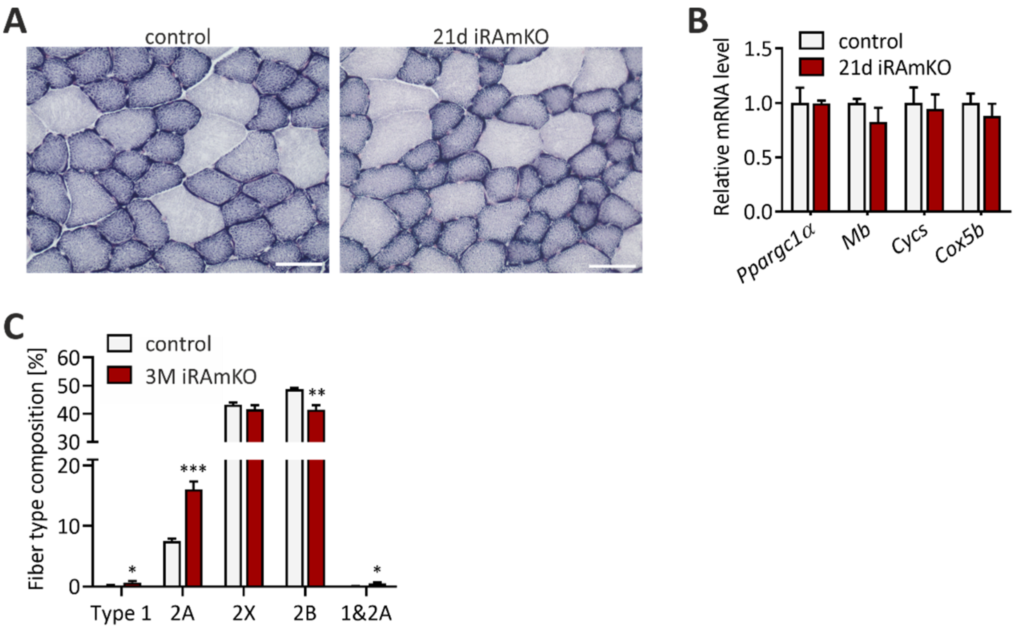

Figure S3. (A) NADH-TR staining of TA cross-sections from 21-day iRAmKOs. (B) Relative mRNA levels of *Ppargc1α* (PGC1α), *Mb* (myoglobin), *Cycs* (cytochrome c) and *Cox5b* (cytochrome c oxidase subunit 5B) in the *soleus* muscle of 21-day iRAmKOs. *C*) Fiber type composition of the TA from 3-month iRAmKOs. n = 4 for 21d iRAmKOs and n ≥ 3 for 3M iRAmKOs. Values represent the mean ± SEM. Significance was assessed using two-tailed unpaired student's *t*-test: *p < 0.05, **p < 0.01, ***p < 0.001.

Figure S4

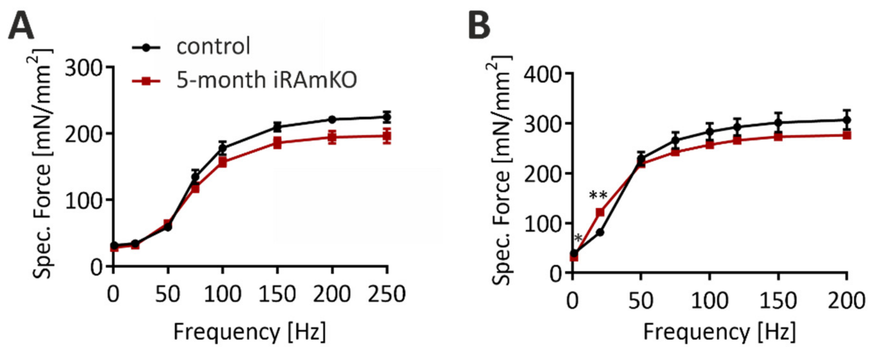

Figure S4. (A) and (B) Specific *ex vivo* muscle force (normalised to the muscle size as explained in material and methods) of the EDL (A) and the *soleus* (B) of 5-month iRAmKOs and controls. 5-month iRAmKOs and controls n = 4. Values represent the mean ± SEM. Significance was assessed using two-tailed unpaired student's *t*-test: *p < 0.05, **p < 0.01, ***p < 0.001.

Table S1

Protein	10 days iRAmKO		21 days iRAmKO		3 months iRAmKO	
	control	iRAmKO	control	iRAmKO	control	iRAmKO
Raptor	1±0.15	0.30±0.02***	1±0.13	0.11±0.03***	1±0.32	0.15±0.06**
mTOR	1±0.06	0.64±0.02***	1±0.14	0.39±0.05***	1±0.15	0.63±0.07*
P-mTOR^{S2448}	1±0.04	0.73±0.01***	1±0.16	0.56±0.04*	1±0.28	0.52±0.09*
P-mTOR^{S2448}/mTOR	1±0.09	1.13±0.04	1±0.05	1.38±0.20	1±0.16	0.86±0.16
4E-BP1	1±0.15	1.26±0.20	1±0.17	1.17±0.29	1±0.06	1.45±0.13**
P-4E-BP1^{S65}	1±0.24	1.73±0.43*	1±0.13	0.64±0.12	1±0.12	0.16±0.04***
P-4E-BP1^{S65}/4E-BP1	1±0.25	1.37±0.34	1±0.17	0.51±0.15**	1±0.12	0.11±0.02***
S6	1±0.04	1.21±0.07**	1±0.20	1.08±0.23	1±0.13	1.01±0.15
P-S6$^{S240/244}$	1±0.14	1.15±0.16	1±0.42	0.87±0.51	1±0.15	0.71±0.13
P-S6$^{S240/244}$/S6	1±0.10	0.95±0.13	1±0.66	0.62±0.25	1±0.25	0.68±0.02
AKT	1±0.07	1.56±0.06***	1±0.20	1.05±0.25	1±0.25	1.42±0.27
P-AKT^{T308}	1±0.14	1.86±0.13***	1±0.16	3.84±2.68	1±0.26	7.67±3.11*
P-AKT^{S473}	1±0.12	1.83±0.39*	1±0.14	3.37±2.04*	1±0.32	5.19±1.76*
P-AKT^{T308}/AKT	1±0.20	1.18±0.13	1±0.28	3.96±3.10	1±0.07	5.18±1.29**
P-AKT^{S473}/AKT	1±0.15	1.18±0.30	1±0.25	2.84±1.08*	1±0.12	3.59±0.63**

Table S1: All quantifications of the western blots shown in Fig. 1C. All values were normalised on α-actinin and on control samples. 10 and 21-day iRAmKOs n = 4, 3-month iRAmKOs n ≥ 3. Values represent the mean ± SD. Significance was assessed using two-tailed unpaired student's *t*-test: *p < 0.05, **p < 0.01, ***p < 0.001.

Table S2

Protein	Heart		Skeletal muscle (TA)	
	control	iRAmKO	control	iRAmKO
Raptor	1±0.24	0.95±0.03	1±0.32	0.15±0.06**
GFP	1±0.28	6.00±3.61	1±0.49	23.15±3.44***

Table S2. All quantifications of the western blots shown in Figure S1B. All values were normalised on α-actinin and on control samples. 3-month iRAmKOs n ≥ 3. Values represent the mean ± SD. Significance was assessed using two-tailed unpaired student's t-test: *$p < 0.05$, **$p < 0.01$, ***$p < 0.001$.

INDIVIDUAL POLYSOME PROFILES

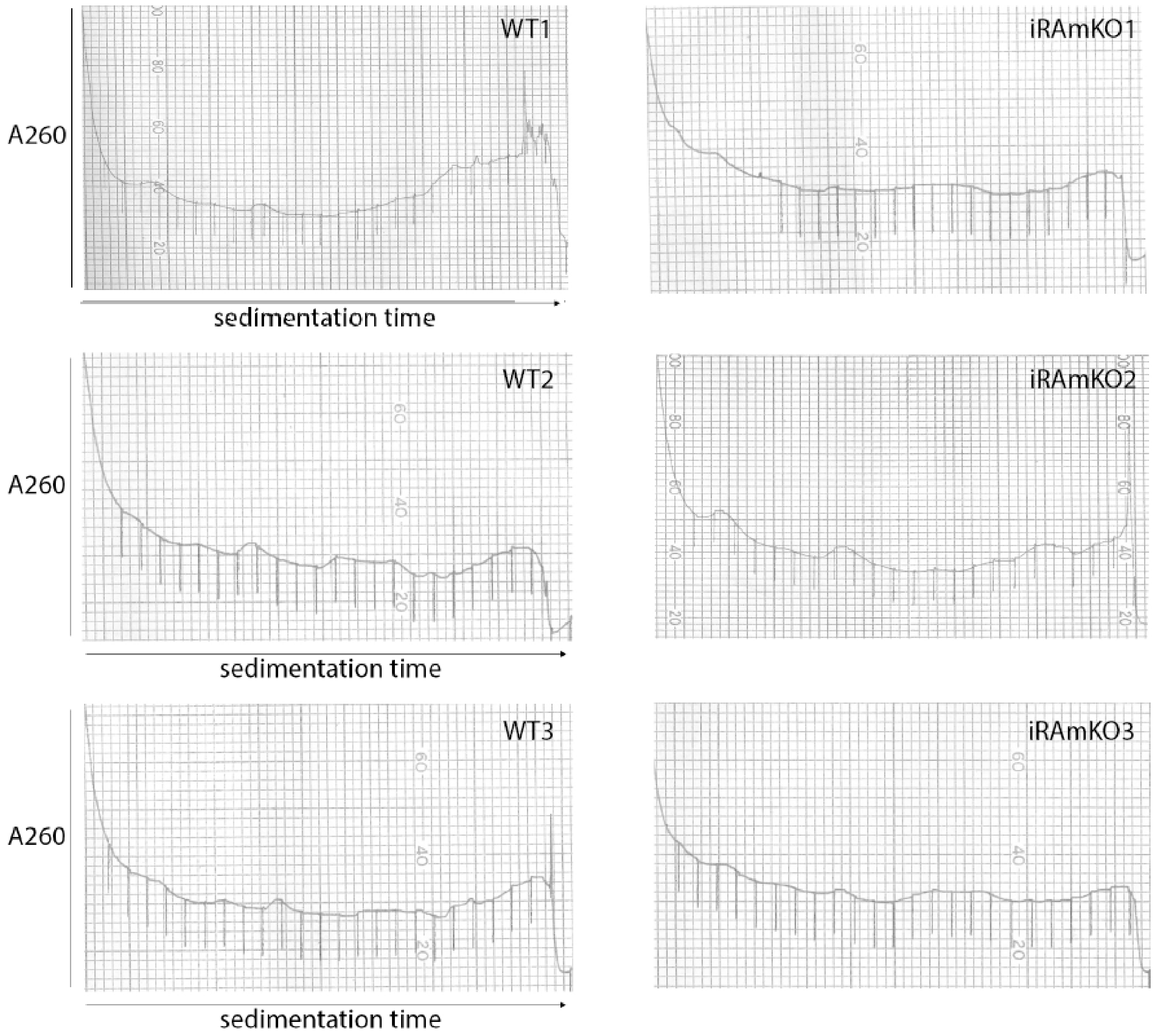

Supplement: Supplementary file 1 — Figure S1. (A) Timeline of TAM injections. (B) Western blot analysis of heart and TA lysates using antibodies directed against the proteins indicated. Note that despite low levels of EGFP expressed in the heart, raptor levels were unchanged, suggesting minimal expression of Cre in this tissue. EGFP was highly expressed in the tibialis anterior (TA) muscle and raptor was strongly reduced. Figure S2. (A) H&E staining of TA cross‐sections from 21‐day iRAmKOs depicted no changes. Scale bar = 50 μm. (B) The variance coefficient using the minimal feret diameter showed an increase in fibre size distribution for type 2A fibers in the TA of 3‐months iRAmKOs. (C) All fiber types of the TA together, however presented no changes in the variance coefficient. (D) Fiber size distribution of type 2A, 2X and 2B fibers from the TA of 3‐months iRAmKOs. (E) Average cross‐sectional area (CSA) of all fibers and (F) average CSA of individual fiber types from the TA of 3‐month iRAmKOs G) average minimal fiber feret diameter (f. f. d.) of all fibers from the TA of 3‐month iRAmKOs and their control littermates. (H) Average CSA and (I) average minimal f. f. d. of all fibers in the TA and soleus (Sol) of 5‐month iRAmKOs. (J) Variance coefficient of individual fiber types in the TA and (K) soleus. (L) Fiber size distribution of type 2A fibers of the TA from 5‐month iRAmKOs. n ≥ 3 (3M) and n = 4 (5M). Values represent the mean ± SEM. Significance was assessed using two‐tailed unpaired student's t‐test: *p < 0.05, **p < 0.01, ***p < 0.001. Figure S3. (A) NADH‐TR staining of TA cross‐sections from 21‐day iRAmKOs. (B) Relative mRNA levels of Ppargc1α (PGC1α), Mb (myoglobin), Cycs (cytochrome c) and Cox5b (cytochrome c oxidase subunit 5B) in the soleus muscle of 21‐day iRAmKOs. C) Fiber type composition of the TA from 3‐month iRAmKOs. n = 4 for 21d iRAmKOs and n ≥ 3 for 3M iRAmKOs. Values represent the mean ± SEM. Significance was assessed using two‐tailed unpaired student's t‐test: *p < 0.05, **p < [file JCSM-11-259-s001.pdf]
